# An Overview of the Application of Viruses to Biotechnology

**DOI:** 10.3390/v13102073

**Published:** 2021-10-14

**Authors:** Carla Varanda, Maria do Rosário Félix, Maria Doroteia Campos, Patrick Materatski

**Affiliations:** 1 MED–Mediterranean Institute for Agriculture, Environment and Development, Instituto de Investigação e Formação Avançada, Universidade de Évora, Pólo da Mitra, Ap. 94, 7006-554 Évora, Portugal; mdcc@uevora.pt; 2 MED–Mediterranean Institute for Agriculture, Environment and Development & Departamento de Fitotecnia, Escola de Ciências e Tecnologia, Universidade de Évora, Pólo da Mitra, Ap. 94, 7006-554 Évora, Portugal; mrff@uevora.pt

**Keywords:** gene expression, gene therapy, nanotechnology, vaccines, viral vectors

## Abstract

Viruses may cause devastating diseases in several organisms; however, they are simple systems that can be manipulated to be beneficial and useful for many purposes in different areas. In medicine, viruses have been used for a long time in vaccines and are now being used as vectors to carry materials for the treatment of diseases, such as cancer, being able to target specific cells. In agriculture, viruses are being studied to introduce desirable characteristics in plants or render resistance to biotic and abiotic stresses. Viruses have been exploited in nanotechnology for the deposition of specific metals and have been shown to be of great benefit to nanomaterial production. They can also be used for different applications in pharmacology, cosmetics, electronics, and other industries. Thus, viruses are no longer only seen as enemies. They have shown enormous potential, covering several important areas in our lives, and they are making our lives easier and better. Although viruses have already proven their potential, there is still a long road ahead. This prompt us to propose this theme in the Special Issue “The application of viruses to biotechnology”. We believe that the articles gathered here highlight recent significant advances in the use of viruses in several fields, contributing to the current knowledge on virus applications.

## 1. Introduction

Viruses are microscopic agents that exist worldwide and are present in humans, animals, plants, and other living organisms in which they can cause devastating diseases. In agriculture, viral diseases may cause losses in all crop yield or be responsible for a drastic decrease in product quality, threatening not only world population nourishment but also the production of fibers, ornamental plants, and medicinal products essential for mankind [1]. In medicine, viruses, such as the ones causing smallpox, influenza, and AIDS, have left their mark on human history, as will also be the case of COVID-19. Therefore, it is not a surprise that viruses do not have the best public image and are fairly seen as our enemies.

The advances of biotechnology and next-generation sequencing technologies have accelerated novel virus discovery, identification, sequencing, and manipulation, showing that they present unique characteristics that place them as valuable tools for a wide variety of biotechnological applications [2]. Viruses possess geometrically sophisticated architectures that make them attractive for materials science and nanotechnology. In addition, they present an efficient machinery and a comprehensive genome structure, which make them easy to manipulate.

Despite this recent technological developments, we can go back to the 18th century to find the first reports of the use of viruses as beneficial, with the first vaccine used against smallpox [3]. By then, it was verified that milkmaids who, due to their close work with cows, contracted cowpox, a cow disease that caused only mild symptoms in humans, and were immune to the human form of the disease, smallpox. After these observations, in 1796, Edward Jenner developed an experiment consisting of scratching a cowpox pustule into the arm of a child who was then exposed to human smallpox virus and revealed to be immune to human smallpox. In the following years, thousands of people were vaccinated by the same way. Of course, not every human disease has an animal analog that confers immunity, and research evolved by developing alternative methods that included the use of disarmed viruses to activate immune system in a putative later virus infection. This was the case of the vaccine against rabies developed by the end of the 19th century by Louis Pasteur as well as vaccines developed later for measles, rubeola, influenza, and polio, to name a few. With the advances of science, new vaccines have been developed with no need to use the entire viral particles and using specific viral proteins instead, overcoming the possible undesirable effect of viral vaccines to cause the disease by themselves. At present, there are extraordinary methods for the production of vaccines. Viral genomes have become easily decodable, and researchers have developed vaccines that rely on the injection of viral RNA into humans so that humans produce viral proteins and activate the immune system. This is the case of the latest vaccines developed for COVID-19 [4]. It is expected, as novel viruses continue to emerge, novel technologies and strategies also continue to arise.

However, vaccines are only one of the many examples of how viruses can be used as beneficial agents. Viruses are involved in many biological processes that have revolutionized some areas, namely gene editing, whose impact is patent through the recent Nobel prize in Chemistry in 2020, attributed to Emmanuelle Carpentier and Jennifer Doudna, for the discovery of tracrRNA as part of the bacteria’s ancient immune system, CRISPR/Cas, that disarms viruses by cleaving their DNA and that has already been applied in several areas as a methodology for highly precise changes in genes [5].

Among viruses, the ones that infect bacteria, the bacteriophages, have also been investigated for their potential to be used in therapies, as they are able to target, infect, and destroy specific bacteria and are harmless to humans [6]. Bacteriophages present a very interesting potential, especially to be used against highly antibiotic-resistant bacteria, one of the biggest public health challenges. In addition, bacteriophages have contributed to biotechnology with many important proteins.

Viruses can also be used as vectors by essentially removing their pathogenic parts while retaining their gene-delivery capacities, making them incredibly versatile tools to carry and deliver genetic material. Viral vectors have been used in gene therapy, i.e., for the introduction of functioning genes into human cells. There are several types of viral vectors used in mammalian cells, including lentivirus, adenovirus, and, the most used virus vectors in gene therapy, the adeno-associated virus (AAV). One example of their use is Luxturna, a gene therapy product, approved in the EU since 2018, that uses AAV to deliver a functional copy of a genetically mutated gene into retinal cells and restore vision of patients with progressive vision loss due to that specific gene mutation. Another example of the use of viruses for gene therapy is to treat cancer, as viruses are able to target and specifically infect cancer cells without harming healthy cells and making tumors more visible to immune system.

In addition to all these biotechnological applications focusing medicine and pharmaceutical industry, viruses have also been used in other fields, such as the agriculture and materials industry. Many plant viruses have been developed as vectors for either the expression and production of a specific protein or for silencing by down regulating the expression of a homologous gene leading to a loss of function, also called virus-induced gene silencing (VIGS) [7,8]. Many applications of viruses have been used for agricultural purposes, namely concerning plant breeding and plant protection. Nevertheless, it is interesting to mention that plants have also many advantages to be used in vaccine production, such as the low cost and low risks they entail, showing once more the versatility of the use of viruses in biotechnology.

Although it will obviously never be ignored that viruses are responsible for devastating diseases, it is clear that the more they are studied, the more possibilities they offer to us. They are now on the front line of the most revolutionizing techniques in several fields, providing advances that would not be possible without their existence. 

## 2. Special Issue Overview

This Special Issue of Viruses, “The Application of Viruses to Biotechnology”, contains eight original articles and seven reviews that demonstrate the current work developed using viruses in biotechnology. These articles were brought by experts that focus on the development and applications of many viruses in several fields, such as agriculture, the pharmaceutical industry, and medicine.

Bacteriophages, the natural predators of bacteria, besides being a promising alternative to antibiotics and especially so for many antibiotic resistant bacteria, are among the many viruses used in biotechnology, mostly due to their easy manipulation and to the many valuable enzymes they possess, such as DNA polymerases and ligases, as well as lytic enzymes that degrade bacterial cell walls and with high potential for phage therapy [9,10]. The study presented by Arsin et al. (2021) allowed the identification and description of a novel marine prophage found in the gram-negative bacterium *Hypnocyclicus thermotrophus*, with similarities to phages infecting gram-positive bacteria [11]. Authors identified a set of nine genes with putative functions, such as cell and nucleotide lysis and replication, interesting for potential biotechnology applications. In addition, the study by Fong et al. (2019) also allowed the isolation and characterization of several phages of the human pathogen *Salmonella enterica*, showing the high phage diversity and phage–host interactions, providing valuable results for the advance on the developing phage-based applications in sectors such as biocontrol [12].

Adeno-associated virus (AAV) vectors are the top platform for gene delivery for the treatment of many human diseases. The review presented by Bower et al. (2021) summarizes recombinant AAV approaches for the treatment of many types of cancer, including interactions with the cellular host machinery to contribute to the enhancement of current strategies to treat cancer. The research articles by Yu et al. (2021) and Yu et al. (2020) presented in this special issue give great contributions for the development of new gene therapy protocols using AAV vectors and provide significant advances for high-level transgene expression from AAV vectors. Yu et al. (2021) showed that, in opposition to what happens with transgene expression using adenoviral vectors, pretreatment with clodronate liposomes does not appear to improve AAV-mediated gene delivery; in fact, it seems to have the opposite effect [13]. In a different study, Yu et al. (2020) demonstrated that an intragenic transcriptional enhancer element within the 3′ end of the env gene of Jaagsiekte sheep retrovirus (JSRV) may be used as a promoter capable of directing transgene expression from AAV vectors in a variety of tissues, particularly liver and lung [14]. These authors also report that these promoter cassettes are small in size, which is suitable for genome constraints of the AAV vector systems. 

Baculovirus expression vectors have been used for the commercial production of complex glycoproteins in eukaryotic cells. Genome engineering of single-copy baculovirus infectious clones (bacmids) has been valuable for the study of baculovirus biology. However, despite their potential, bacmids are not yet widely applied as expression vectors mainly due to the easy loss of gene-of-interest (GOI) expression. The study by Pijlman et al. (2020) revealed that the relocation of the attTn7 transgene insertion site away from the mini-F replicon in single-copy baculovirus infectious clones prevents deletion of the gene of interest, thereby resulting in higher and prolonged recombinant protein expression levels [15]. In this work, the authors were able to use a novel bacmid to produce chikungunya virus-like particles for industrial vaccines. This study shows great advances on the use of bacmids as expression vectors, whose limitation is the rapid loss of GOI expression. Hsu et al. (2020) optimized a polycistronic baculovirus expression vector to express virus-like particles (VLPs) containing several portions of *Porcine epidemic diarrhea virus* (PEDV) to elicit immunity against PEDV in pigs [16]. These authors also verified that pigs immunized with VLPs together with a mucosal adjuvant showed a higher protection against PEDV, which is of great interest for the development of other enteric viral vaccines.

The lactic acid bacterium *Lactococcus lactis* is widely used in dairy fermentation and is considered safe to be used as host for biopharmaceutical development [17]. In the study presented in this special issue by Wang et al. (2020), authors constructed a recombinant *Lactococcus lactis* expressing a protein of the novel variant of the infectious bursal disease virus (IBDV), against which the current conventional IBDV vaccine cannot completely protect [18]. With this new vaccine, they were able to immunize chickens, which produced unique, neutralizing antibodies and provide results showing the potential of *L. lactis* in vaccine development.

Among the many viruses that have been used as vaccine vectors is the Newcastle disease virus (NDV), in the review presented by Bello et al. (2020), the molecular biology and approaches to engineer NDV into an efficient vaccine vector is discussed, focusing on the prospects of the virus as a vehicle of vaccines against cancer and infectious diseases in humans and animals [19].

The study by Maeda et al. (2020) gives important contributions on the use of viruses to accelerate plant breeding. Authors used *Apple latent spherical virus* (ALSV) to induce flowering in grapevine (virus-induced flowering, VIF) by expression of the Arabidopsis flowering locus T gene; this study shows the potential of ALSV vectors as VIF to shorten the generation time of grapevine seedlings [20].

This special issue also covered aspects concerning the use of viruses against the new disease that changed the world in the recent years, COVID-19, demonstrating how viruses can be rapidly redirected to fight against a new threat. In the review by Lundstrom (2020), the advantages of using viral particles and RNA replicons and DNA replicon vectors of RNA viruses for vaccine development are presented, with a special emphasis on COVID-19 viral-based vaccines [21]. The review by Fernandez-Garcia et al. (2020) summarizes the recent research on viruses for therapy and diagnosis of COVID-19, namely viral-vector vaccines, bacteriophages to find SARS-CoV-2 antibodies, and their use as a treatment [22].

The latest progress on the development of viruses as vectors in biotechnology and their many applications, such as molecular breeding, functional genomic studies, and vaccines, are reviewed by Wang et al. (2020). These authors summarize available viral vectors for economically important crops [23].

Virus-like particles (VLPs) and virus nanoparticles (VNPs) are increasingly being used for a variety of applications in biotechnology. In the review by Venkataraman and Hefferon (2021), the use of *Tobacco mosaic virus* (TMV), *Potato virus X* (PVX), *Cowpea mosaic virus* (CPMV), and geminiviruses for biotechnological purposes is discussed with a great focus on the major achievements of these viruses as expression vectors in medicine and human health [24].

We also had the privilege to contribute to this special issue by providing a review where we focused on the advances on the CRISPR technology to target viruses and achieve plant viral resistance but also, and in line with this special issue, the use of viruses as vectors for CRISPR technology, discussing the advantages and disadvantages of their use as alternatives to other platforms. It is interesting to mention that during the preparation of the review, an increasing number of studies concerning the use of different and new viruses were constantly being published, and a great effort was made to keep the review as up to date as possible, showing the continuous, growing applications of the use of viruses in biotechnology [25].
